# Taxonomic variation in immune response strategies among primates

**DOI:** 10.1038/s41435-025-00363-1

**Published:** 2025-10-23

**Authors:** Sophie K. Joseph, Jordan M. Lucore, John Lindo, Elizabeth V. Lonsdorf, Marcela E. Benítez

**Affiliations:** 1 Department of Anthropology, Emory University, Atlanta, GA USA; 2 Department of Anthropology, University of Michigan, Ann Arbor, MI USA; 3 Department of Molecular and Cell Biology, University of California, Berkeley, CA USA

**Keywords:** Genome evolution, Adaptive immunity, Innate immunity, Genetic variation

## Abstract

In primates, mechanisms of both innate and adaptive immunity are well-studied in just a few species out of this very diverse taxonomic order, especially in relation to specific pathogens like SIV. Recent research has indicated there may be taxon-specific differences across primates in immune response strategies, including relative proportions of immune cell types and whether adaptive or innate responses are favored. It remains unclear which taxonomic level best explains variation in primate immune response strategies. Identifying this is important for understanding when and why these differences evolved. This review synthesizes major recent findings in primate immunology, to connect them to more generalized research on immune response strategies and present hypotheses for future research, focusing on major methodologies in the field. We demonstrate that gaining a better understanding of the evolution of primate immunity has far-reaching implications for our understanding of the evolutionary past of humans, and for present-day global health.

## Introduction

Cellular responses that recognize and neutralize invading pathogens are an important part of survival and maintaining homeostasis—this is the basic principle of innate immunity [1]. Gnathostomes, or jawed vertebrates, are the only known animals to also have evolved adaptive immunity, which uses immunoglobulins and T-cell receptors to launch an antigen-specific response [2]. Understanding the evolution of immunity within our branch of vertebrates, the taxonomic order Primates, is a critical context for understanding many aspects of human immunity, health, and disease. In non-human primates (hereafter *primates*), the mechanisms of both innate and adaptive immunity are relatively well-studied, especially in relation to specific pathogens like **simian/human immunodeficiency virus (SIV/HIV)** (see Glossary) [3–8]. The majority of what we know about primate immunity comes from a few primate species, mainly chimpanzees and macaques, which have been the subject of biomedical testing for decades. However, there are now almost 500 named primate species, with tremendous ecological, geographic, and phylogenetic diversity [9, 10]. In the last decade, due in large part to the development of novel methods to examine immune responses in a variety of primate species, we are beginning to generate a richer knowledge of the varied ways in which primates respond to pathogens and disease, and the factors that may influence those strategies. For example, recent research suggests that primates may differ widely in how their immune system responds to a threat, such as in the types and proportions of immune cells they produce and in how much they rely on adaptive versus innate immune responses [11–17]. Such findings highlight the need for a diverse approach to studying immunity across the primate taxa, because understanding this could shed light on when and why these differences evolved in the evolutionary past.

This review will synthesize major pathogen-specific findings in immunity across primates, in order to connect these findings to more generalized research on immune response strategies and present hypotheses for future research in this area. We begin with a short and accessible section on the molecular mechanisms of innate and adaptive immunity in primates. We then review important work in the study of primate immunology, focusing on major methodologies in the field. We consider these methods in three main groups: Variation in Cell-Surface Receptors, Primate Functional Genomics, and Characterizing Systemic Immune Responses Using Inflammatory Biomarkers. Each set of methods offer valuable insight into how primate immunity varies and segregates across taxonomic lines across different species. We conclude this review with a call for a more holistic approach to the study of immunology in nonhuman primates. We posit that merging our knowledge of life history and primate behavior with the growing knowledge of taxonomic differences in immune response will help guide primate evolutionary biology and immunology in new and exciting directions.

## Innate and adaptive immunity in primates

**Fig. 1:**
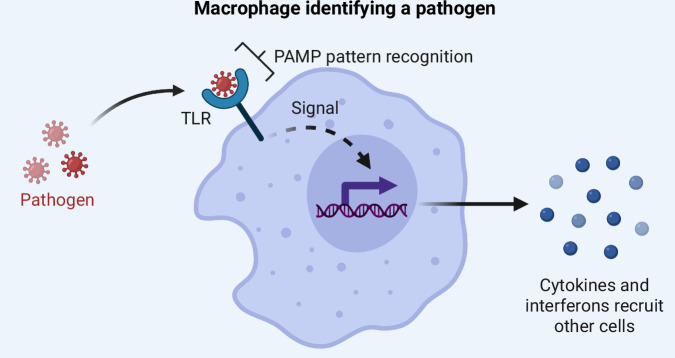
Illustration of macrophage identifying a pathogen. Macrophages use TLRs to recognize pathogens and then recruit other immune cells by releasing cytokines and interferons. Created with BioRender.

We begin this review with a brief discussion of molecular mechanisms of innate and adaptive immunity in primates. Innate immunity recognizes and destroys invading pathogens by reading molecular patterns on non-host cells. It is the primary line of defense against invaders and begins almost immediately upon a pathogen entering the body [1, 18]. Neutrophils and macrophages are the most abundant innate effector cells, and natural killer lymphocytes also play an important role. Macrophages—large cells which engulf pathogens—utilize several cell surface receptors to identify foreign molecules. This includes **toll-like receptors (TLRs)**, particularly important to understanding human evolution in the larger context of primate immunity because they have been under positive selection in our great ape lineage, which includes humans [11, 12, 19]. TLRs are cell-surface ligands that recognize **pathogen associated molecular patterns (PAMPs)**, common markers of bacterial, viral, macroparasitic, and protozoan molecules [1]. Following this initial pattern recognition, macrophages release chemical signals to recruit other immune cells (Fig. 1).

Neutrophils, which represent over 50% of circulating immune cells in humans—a larger proportion than is found in other primates—are recruited to the site of infection by chemical signals from macrophages and infected endothelial cells lining the tissue [15, 20, 21]. Unlike macrophages, which live for several weeks, neutrophils are usually only functional for 1-2 days and work primarily within the blood circulation instead of within the tissue itself. Current research is aimed at understanding why humans produce higher levels of these short-lived, energetically expensive immune cells, even compared to other great apes [15, 21]. Neutrophils recognize and neutralize invaders by using cell-surface TLRs and then engulfing the foreign cell. In addition to engulfing pathogens, neutrophils can also cast extracellular nets to trap pathogens and produce reactive oxygen species, which are lethal to invaders [20].

**Fig. 2:**
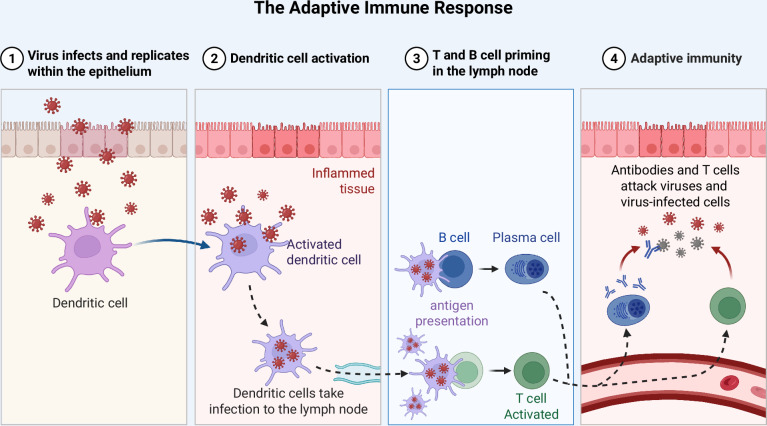
Illustration detailing adaptive immune response. This includes antigen presentation by activated dendritic cells, training cytotoxic T-cells, and release of antibodies by plasma cells. Created with BioRender.

Rather than simply reacting to a set of common foreign molecular patterns, the adaptive immune response—found only in jawed vertebrates—trains immune cells to target cell-surface molecules for each unique pathogen. This provides an advantage for the host when a pathogen may not display typical molecular patterns, or is otherwise evading the innate immune system. This training is a trade-off, because the adaptive response takes days, not minutes, to mount [18]. Nonetheless, this tailored response to unique cell-surface molecules, or antigens, has helped jawed vertebrates to inhabit a wide array of environments across the globe with high microbial variability. The main effector cells in primates are T-cells and B-cells, of which there are many subtypes. In general, T-cells are the cytotoxic effectors which kill pathogens and infected cells that display the antigen. B-cells differentiate into plasma cells, which secrete antibodies that bind to the antigen and kill the pathogen (Fig. 2). Both T- and B-cells are activated by antigen presenting cells, most commonly dendritic cells [2, 18].

## Variation in cell-surface receptors

The study of immunology in primates is methodologically diverse and quickly changing, especially with respect to phylogenetic analyses for detecting natural selection on genes coding for a specific immune cell-surface receptor. Because this review is concerned with taxonomic differences among primates, we pay special attention to the relevance of these methods to broader phylogenetic understanding. Phylogenetic analysis provides valuable insight into when and in which common ancestor a genetic mutation occurred in the primate lineage. This becomes particularly relevant in the case of important cell-surface immune cell receptors, which are at the forefront of the host-pathogen interface. Variation in cell-surface molecules first became a focal point of primate immunology via research on SIV/HIV, because of the potential implications for human HIV treatment and prevention. We therefore discuss important examples of cell-surface receptor diversity in the context of this research, as well as expand beyond the context SIV/HIV.

**Fig. 3:**
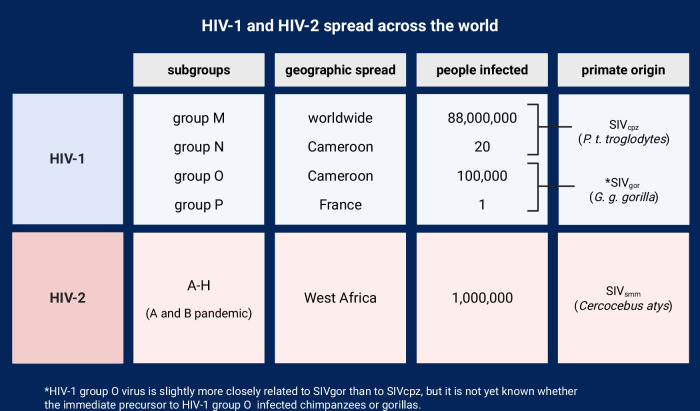
Summary of current knowledge on HIV-1 and HIV-2 spread across the world. Contains information on each viral subgroup’s primate origins, geographic spread in humans, and approximate number of humans infected [3–8]. Created with BioRender.

More than 40 species of primates have documented species-specific types of SIV, which is known as HIV in humans [8]. These viruses attack and replicate within helper T-cells. In a select few primates, SIV can cause progression to the fatal **acquired immunodeficiency syndrome (AIDS)**. Curiously, in most other primates, progression of SIV to AIDS is not observed, meaning that these primates appear to live with SIV infection without detrimental health effects [3, 6]. This has centered non-human primates as critically important for research on treating and curing HIV, creating one of the most long-standing research traditions within primate immunology. A decade into the **HIV-1 group M** pandemic, the identification of the SIV lineages most closely related to HIV-1 and HIV-2 were—and arguably remain today—some of the most seminal works in primate immunology [5, 7]. These studies primarily utilized proviral DNA, the genetic footprint of the virus trying to replicate in human cells. Today, studies of SIV viruses in primates instead directly use viral RNA from fecal samples to analyze the viral genome more directly. This has led to some important new discoveries. For example, the origin of HIV-1 group O was originally attributed to a strain of SIV_cpz_ based on a proviral DNA phylogenetic analysis [5]. However, using fecal RNA, its origin is now attributed to SIV_gor_ in western lowland gorillas, not chimpanzees (Fig 3) [8].

Most non-human primates infected with SIV, the primate version of HIV, do not develop AIDS, whereas humans almost always do. Researchers are still exploring why this is, and it is a complex question, as an AIDS-like illness has been documented in some wild chimpanzees of Gombe National Park, Tanzania [22, 23]. This means that tolerance of SIV infection is not universal among non-human primates, either. This further highlights the need to study a wide range of primate species to understand the diversity of immune defenses. For example, some species like chimpanzees and certain African monkeys may avoid AIDS because they retain more helper T-cells and show lower inflammation during chronic infection [3, 6]. Recent research suggests that greater genetic diversity in the CD4 protein on helper T-cells may help block the virus from entering these cells, keeping their viral burden low [24]. Another cell-surface receptor, **Tripartite Motif-containing protein 5 (TRIM5)**, is a possible mechanism for why pandemic HIV-1 has spread to over 88 million humans [25, 26]. Studies of the pandemic strain HIV-1 (known as group M) in humans and other primate cell lines suggest that this strain of HIV-1 is better able to evade the anti-viral effects of TRIM5 than other strains [25]. The *TRIM5* gene varies considerably among humans, other apes, and monkeys, with the neotropical and African and Asian monkey variants of the TRIM5 receptor providing the most potent protection against HIV-1 infection in helper T-cells [27].

The **Major Histocompatibility Complex (MHC)** represents another important location of variation among primate taxa—in fact, it is one of the most polymorphic gene families in existence among primates [28]. The MHC codes for cell-surface proteins that bind to fragments derived from pathogens, which are displayed on a cell’s surface to be recognized by T-cell receptors [29]. Here, we discuss MHC Class I and Class II, the most polymorphic of the MHC genes and directly involved in antigen presentation. While functionally important for the cytokine-driven inflammatory response, Class III molecules are largely conserved among primates, and even mammals, making them less relevant in the context of taxonomic variability [30]. MHC Class I molecules are found on the surface of most cells within the body and have roles in innate immunity, adaptive immunity, and placenta formation in reproduction; MHC Class II molecules are mainly found on the surface of antigen-presenting cells (dendritic cells, macrophages, and B-cells), performing mainly adaptive immune functions. MHC Class I is more rapidly evolving than Class II, likely because of its diverse functions [31]—as a result of this quick evolution, only great apes have strict orthologs of all of the polymorphic human MHC class I genes (known as Human Leukocyte Antigens HLA-A, HLA-B, and HLA-C) [31, 32]. These genes have dozens, sometimes even hundreds, of different alleles present in each species, meaning there is a lot of individual variability.

This variability has functional importance, as it makes a population resilient to more pathogens, since different MHC alleles have different binding specificities for peptide fragments from pathogens. Many HLA-A, -B, and -C alleles can be organized into cross-species clades made up of different ape orthologs. For example, HLA-B Clade 1 has HLA-B and the primate orthologs like chimpanzee (*Pan troglodytes)* Patr-B, bonobo (*Pan paniscus)* Papa-B, Gorilla (*Gorilla gorilla*) Gogo-B, etc. In particular, -B Clade 1 contains the HLA-B*57:01 allele and the chimpanzee Patr-B*06:03 allele, both functionally associated with control of the progression of HIV-1 and SIVcpz infection, respectively [33]—yet, Papa-B Clade 1 alleles have not been identified in bonobos, despite that this clade is predicted to have been present in the common ancestor of chimpanzees and bonobos [31]. This suggests it may have been lost in the bonobo lineage, or at least driven to very low frequency. Unlike chimpanzees and humans, bonobos don’t seem to have a SIV circulating in their populations [34], a potential contributing factor to the loss of this allele.

Though cell-surface receptor diversity first gained attention in the context of SIV/HIV, immune defenses among primates are shaped by diverse genes that influence how cells recognize and respond to a variety of other pathogen threats. Natural killer cells, part of the innate immune system, use specific receptors called **Killer Immunoglobulin-like Receptors (KIR)** to identify and avoid attacking their own cells. The genes that control these receptors are particularly diverse in monkeys and apes, suggesting they have evolved unique immune strategies compared to other mammals [16]. Similarly, primates have distinct versions of **Interferon-induced Transmembrane proteins (IFITM)** genes, which help block viruses from entering cells. All apes share a specific arrangement of *IFITM* genes, which likely emerged after the ape lineage split from other primates [35, 36]. These examples show that primates have evolved varied immune strategies, with unique genetic adaptations in monkeys and apes that may help explain differences in susceptibility to diseases. Understanding this diversity could provide insights into why certain species are more resistant to infections and improve our understanding of immune responses across the primate order.

As the first line of defense for identifying pathogens, the extracellular domains of toll-like receptors (TLRs) constantly interface with the cell-surface receptors of pathogens. Thus, it stands to reason there should be an evolutionary race between TLRs for improving pattern recognition (and the pathogen’s evasion of this recognition) [11, 12]. While several studies examining nucleotide substitution rates through evolutionary time in primates have detected positive selection on the majority of the TLR genes, *TLR4* in particular consistently displays the strongest signature of selection across several studies with different sized datasets of primate species [11, 12, 19]. One study found the extracellular domain of the cell-surface protein TLR4 was under particularly strong positive selection in the lineage of all great apes, and again in the gorilla lineage after its split from other great apes [19]. Another similar study, which looked at a larger region of the TLR genes beyond just the extracellular domains, identified the strongest positive selection to be in *TLR4*, within the lineage shared by African and Asian monkeys and apes [12]. TLR4 is the toll-like receptor responsible for recognizing *Trypanosoma* protozoans and the molecular footprint of bacterial lipopolysaccharide (a protein found on Gram-negative bacteria) [11, 12, 19]. It is reasonable that the repeated interaction of a pathogen with a host primate’s TLRs would provide this variety of selective pressures, since each TLR is responsible for recognizing a different set of molecular patterns unique to each different pathogen type (e.g., bacteria, viruses, parasites, etc.). For example, the particularly strong positive selection on *TLR4* may indicate that Gram-negative bacteria were especially important selective pressures in the African and Asian monkey and ape lineages [11, 12, 19, 37]. Functional genomics research has made evaluating such phylogenetic hypotheses possible, making it critical for studying the real-world implications of taxon-level variation among primates.

## Primate functional genomics

Functional genomics links genes with their expression and downstream phenotypes. Fundamental techniques include RNA-seq and expression microarrays for characterizing genome-wide expression levels in in vitro tissues, at different time points and/or experimental conditions [38]. Additionally, the field is being advanced by new technologies like induced pluripotent stem cells and massively parallel reporter assays. Used in tandem with phylogenetic studies to map where functional changes occurred in evolutionary time can provide evidence regarding the effects of an evolved trait. In the context of primate immunity, this can help answer questions like whether there is more reliance on innate or adaptive immunity in each species, why the relative proportion of immune cells produced differs between species, or what contributes to different levels of systemic inflammation upon invasion by the same pathogen. Understanding what level of taxonomic organization accounts for the most variation could give clues to why these differences evolved in the evolutionary history of primates.

Recent work combining phylogenetic analyses and functional genomics has shed light onto the selective pressure posed by Gram-negative bacteria in the great ape lineage. Further, of relevance to human health specifically, this work includes a potential explanation for why humans in particular have an exceptionally strong immune response to stimulation with the Gram-negative bacterial protein lipopolysaccharide [39, 40]. Even small doses of lipopolysaccharide can induce powerful immune responses in humans and initiate sepsis, an extreme immune host response which leads to tissue damage and organ failure. However, African and Asian monkeys and other apes require doses of lipopolysaccharide that are an order of magnitude higher than humans to induce sepsis to a similar degree [39]. A recent study correlating primate body size to the strength of lipopolysaccharide response across nine primate species (spanning more than three orders of magnitude of body mass) suggests that body size may account for some of this variation. Larger-bodied organisms tended to have an exaggerated innate immune response to lipopolysaccharide stimulation. Specifically, this study measured transcriptional expression of innate immune genes using RNA-seq in response to this stimulation, and found an outsized increase in gene expression levels in the larger organisms [40]. RNA-seq profiles genome-wide expression levels using next-generation sequencing of transcribed RNA. However, with human and ape body mass represented with just one human and one Sumatran orangutan (*Pongo abelii*) [40], this study likely did not have sufficient power to detect gene expression differences that could account for why apes require doses of lipopolysaccharide an order of magnitude larger than humans to induce sepsis [39]. Thus, more work is needed to determine the relative contributions of body size, taxonomy (within similar body size categories, like humans versus other apes), and other factors affecting interspecies variability.

Tissue culture, a method which allows for the in vitro characterization of the functional impacts of genetic variation in actual primate cells, is a common technique for studying cellular responses in a safer manner than using live organisms. Using a multi-species gene expression microarray, which tracks expression levels of transcribed RNA across the genome, Barreiro and colleagues identified 793 genes that were highly expressed in tissues from human, chimpanzees, and rhesus macaques after stimulation in vitro with bacterial lipopolysaccharide molecules. Many of these genes were involved in the TLR4 pathogen recognition response [41]. Similarly, a study of the **CEACAM3 receptor**, which helps neutrophils recognize dangerous bacteria like *Haemophilus aegyptius* and *Helicobacter pylori*, identified different versions of the *CEACAM3* gene in the lineage shared by apes and African and Asian monkeys, but found it to be absent entirely in neotropical monkeys [42]. As with TLR4 [19], they found the highest level of variability in the extracellular part of the CEACAM3 protein that directly interacts with pathogens to help immune cells recognize them [42]. This extracellular domain also shows statistical evidence of positive natural selection in the ancestor of apes and African and Asian monkeys. Crucially, Adrian and colleagues then studied the binding capabilities (to recognize *H. pylori* bacteria) of these different versions of the CEACAM3 protein, confirming these genetic variants do alter the ability of the different primate taxa to recognize bacterial pathogens. Such studies highlight critical variation in the genes involved with primate immune responses, and also represent a much-needed synthesis between genomic sequence phylogenies and the functional biological consequences of these sequence variations.

Hawash and colleagues characterized immune gene expression in response to both bacteria and viruses, using an in vitro study on innate immune cells from humans, chimpanzees (*Pan troglodytes*), olive baboons (*Papio anubis*), and rhesus macaques (*Macaca mulatta*) [15]. Their analysis centered around splitting these species in two phylogenetic categories: apes vs. African and Asian monkeys. In response to stimulation by both bacterial lipopolysaccharide and gardiquimod (mimics single-stranded RNA viruses), the transcriptional profiles from RNA-seq indicated three times more ligand-specific targeting of either the lipopolysaccharide or gardiquimod conditions by the African and Asian monkeys than by the apes. They found that apes essentially produce a stronger, but less pathogen-specific, immune response than the African and Asian monkeys [15]. Their methodology and phylogenetic framing of results is a promising avenue of future research. A broader sample of taxa would unite functional and phylogenetic approaches and shed light onto evolutionary reasons behind these differences in immune response strategies. Given that neotropical monkeys do not display comparable signatures of selection on *TLR4* to apes and African and Asian Monkeys [11, 12, 19], it would be useful to compare their transcriptional profiles under the lipopolysaccharide condition, which could be informative of the selective pressure posed by Gram-negative bacteria in each lineage.

A significant barrier to expanding species representation is the difficulty of obtaining cell lines for use in functional studies. **Induced pluripotent stem cells (iPSCs)** offer a solution, as they can be differentiated into tissues that are typically difficult to collect from living primates, helping bridge this gap. iPSCs are also renewable, preserving access to these tissues long-term. This is especially valuable for functional studies involving apes, as tissue collection from chimpanzees is no longer permitted in the United States [38]. Other new methods, such as **massively parallel reporter assays (MPRA)**, can simultaneously test how thousands of genome segments affect gene regulatory activity [43]. MPRAs can screen for enhancer function in primate cell lines (enhancers are DNA regions that increase the transcriptional output of target genes), providing a genome-wide picture of transcriptional regulation. This is important in an evolutionary context, as many functional studies primarily involve protein-coding genes. However, non-coding regulatory regions, which make up the majority of primate genomes, are also undoubtedly subject to selective pressures from pathogens and other evolutionary constraints [44].

## Characterizing systemic immune responses using inflammatory biomarkers

There has been a recent push to understand how primate immunity varies across taxonomic lines at the systemic level (i.e., involving multiple immune cell types), beyond characterizing one single type of immune cell receptor. In addition to statistical phylogenetics or functional genomics from in vitro assays, it is important to study immunity in the full context of the body system in live primates. Sampling inflammatory biomarkers (both invasively and non-invasively) are key methods commonly used in studying differences in systemic immune responses among primates and humans, which we explore in this section of the review.

Immune senescence and increased inflammation, two key processes involved in human aging, are promising areas for broader phylogenetic comparisons of immune function. Most research, other than in humans, has occurred in rhesus macaques (*Macaca mulatta*) as representatives of African and Asian monkeys. Two rhesus macaque studies, one focused on immune senescence in innate immune cells like neutrophils, basophils, and monocytes [21], and the other on T-cells and monocytes [13], generally report that rhesus macaques are similar to humans in exhibiting immune senescence at old age [45]. For example, Asquith and colleagues note a similar trend of decreasing naive T-cells and increasing terminally differentiated T-cells with age [13]. Additionally, serum levels of pro-inflammatory **Interleukin-1 receptor antagonist** (**IL-1Ra)** cytokine were positively correlated with age in free-ranging female rhesus macaques at Cayo Santiago, Puerto Rico but showed no relationship with female reproductive status [46]. The similarity in aging patterns between macaques and humans may directly reflect evolutionary trends in immune senescence in haplorrhines, or could instead be a by-product of longer lifespans and other differing life history strategies between strepsirrhines and haplorrhines.

Expanding aging studies beyond macaques has already provided some insight into which other primate groups mirror aging in humans, and whether these differences result from lifespan alone. For example, little is known about strepsirrhine immune senescence with respect to innate immune cells and T-cells, highlighting the importance of expanding investigation to more species in this context. However, a few strepsirrhine species have been considered in other aging-related processes like inflammation. Unlike in humans and rhesus macaques, a study of age-related inflammation in captive sifakas (*Propithecus coquereli*) and ring-tailed lemurs (*Lemur catta*) found no evidence for age as a predictor of increased inflammatory biomarkers or oxidative damage, despite the different life history strategies of these two strepsirrhines (e.g., sifakas live longer and have later reproductive maturation than lemurs) [47]. This suggests that expanding strepsirrhine studies to include immune senescence of innate immune cells and T-cells would be very revealing of phylogenetic differences in immune senescence across primates, especially between strepsirrhines versus haplorrhines. Furthermore, a framework for studying the socio-environmental influences on aging in a wider sample of haplorrhines beyond humans and macaques has been recently proposed for a species of neotropical monkey, white-faced capuchins (*Cebus imitator*) of Santa Rosa National Park, Costa Rica, given their access to long-term longitudinal data on behavior, biomarkers, and environmental conditions [48]. Monitoring immune function in conjunction with aging and reproductive status could evaluate how life history strategies affect immune function, possibly informing the evolution of taxon-specific phenotypes in these neotropical monkeys and how they differ from apes and African and Asian monkeys or strepsirrhines.

Unfortunately, it is currently difficult both to compare taxon-specific differences in immune function and monitor them longitudinally in a particular species, especially in free-ranging populations located in remote areas. The majority of in vivo research has been conducted on primate populations suitable for invasive biological sample collection (e.g., blood draws) that require capture and anesthetization, which are stressful procedures that can be difficult to safely implement in wild populations, especially in arboreal species [49]. Recently, more research has utilized non-invasive markers of immune function to promote the inclusion of understudied species in immunological research, such as strepsirrhines and neotropical monkeys, and enable functional comparison across primate taxon. Three non-invasive markers of immune function, urinary neopterin, urinary suPAR, and fecal immunoglobulin A (IgA), have been successfully validated in primates and offer promising avenues for non-invasive immunological research in wild primate populations.

Neopterin, a biomarker excreted primarily from innate effector cells, such as macrophages [50], is currently the most widely utilized immune biomarker in primate field studies. Higham and colleagues validated neopterin as a reliable indicator of viral infection in primates and illustrated a strong correlation between serum and urinary neopterin values [51], paving the way for neopterin to be used in non-invasive research. Urinary neopterin has since been validated in a range of taxa (e.g., apes [52], African and Asian monkeys [53], and neotropical primates [54]) and is now considered a reliable marker of immunosenescence [53–55] and a possible indicator of habitat quality [52, 54] and environmental conditions like temperature [56]. Moreover, increases in neopterin levels were correlated with acute outbreaks of respiratory disease in wild chimpanzees (*Pan troglodytes*) [57] and bonobos (*Pan paniscus*) [58], while lower neopterin levels were associated with increased richness of gastrointestinal helminths in wild geladas (*Theropithecus gelada*) [59]. Neopterin levels have also been correlated with infection outcomes in SIV-infected mandrills (*Mandrillus sphinx*) [60]. Taken together, these findings demonstrate the utility of neopterin across studies of generalized immune activation and disease-specific research.

Urinary suPAR is a proinflammatory biomarker and can also be measured noninvasively in urine; however, it has been utilized in fewer studies and has only been validated for use in rhesus macaques. **Urinary suPAR** is the soluble form of the **urokinase plasminogen activator** receptor which is the binding site for the urokinase protein (uPAR), an enzyme found on both innate and adaptive effector cells that plays a crucial role in cell remodeling [61, 62]. Unlike neopterin, which showed minimal response to tissue damage, urinary suPAR was shown to reflect surgically inflicted tissue damage [61], likely making it a useful biomarker for non-invasive monitoring of wound severity and healing. However, urinary suPAR exhibited a weaker response to viral infection than neopterin [61] and did not reflect age-related changes in immune function [63]. Therefore, urinary neopterin and suPAR likely indicate different immune processes and could possibly be used to evaluate taxon-specific differences in immune function to distinct immune perturbations (e.g. viral infection versus wound healing).

Distinct from neopterin and suPAR, **immunoglobulin A (IgA)** is an antigen crucial to mucosal-based immunity that helps protect the epithelium from opportunistic pathogens. Fecal IgA, specifically, represents the amount of secretory IgA in the gastrointestinal tract and has been shown to reflect the function of the intestinal mucosal immune system [64, 65]. In contrast to urinary neopterin and suPAR, which measure systemic immune activation, fecal IgA is a promising biomarker to quantify immune function specific to the gastrointestinal tract which is constantly exposed to toxins and enteric pathogens including intestinal parasites. For example, higher IgA has been associated with lower whipworm parasite egg counts in wild baboons (*Papio cynocephalus*) [66]. Fecal IgA may also be an ideal biomarker to study the relationship between the gastrointestinal microbiome and host immunity to expand our understanding of how microbial communities assist in the development and maintenance of host immune function. Fecal IgA has primarily been used as a marker of stress in captive primates [67, 68] because chronically elevated cortisol levels can cause immunosuppression, decreasing fecal IgA. In wild chimpanzees, fecal IgA was shown to reflect the same seasonal patterns as respiratory illness with higher levels of fecal IgA during the late dry season, which is the period typically associated with higher rates of respiratory infection [69]. However, this study also noted that seasonal changes in nutritional stress and parasite infection could be correlated with IgA concentrations. In addition, Behringer and colleagues caution from their study of Barbary macaques (*Macaca sylvanus*) that the strong responsiveness of fecal IgA to Hypothalamic-Pituitary-Adrenal-axis activity may limit the interpretability of IgA as a measure of immunity [70]. Thus, more work is necessary to determine whether fecal IgA is a suitable biomarker of immune system function in primates. Work expanding species representation through IgA validation studies, as recently done in red fronted lemurs (*Eulemur rufifrons*) [71], will help characterize the immunological correlates of IgA in more primate species.

Our understanding of systemic immune function in primates will benefit greatly from integrative work on senescence, gene expression data, and measures of inflammatory biomarkers. In particular, non-invasive methods for characterizing biomarkers like suPAR, neopterin, and IgA are rapidly improving, and additional markers are continuing to be validated and deployed (Table 1). This is critical for expanding species representation in primate immunity research, as the ecological and geographic diversity of primates presents challenges for using invasive methods, particularly in free ranging populations. In the following section, we outline a framework for future research that is based on the current available data in hopes of generating advancements in this area.

**Table 1:** Summary of currently available studies on suPAR, neopterin, and fecal IgA immune biomarkers in both captive and free ranging primates.

Biomarker	Species	Population	Location	Ref
Neopterin	Bonobo (*Pan paniscus*)	captive	several European zoos	[52]
Neopterin	Chimpanzees (*Pan troglodytes*)	captive	several European zoos	[52]
Neopterin	Barbary macaque (*Macaca sylvanus*)	semi-free ranging	Affenberg Salem, Germany	[53]
Neopterin	Tufted capuchin (*Sapajus apella*)	captive	Language Research Center, Georgia State University, USA	[54]
Neopterin	White-faced capuchin (*Cebus imitator*)	free ranging	Taboga Forest Reserve, Costa Rica	[54]
Neopterin	Chimpanzee *(Pan troglodytes schweinfurthii*)	free ranging	Kibale National Park, Uganda	[55]
Neopterin	White-faced capuchin (*Cebus imitator*)	free ranging	Taboga Forest Reserve, Costa Rica	[56]
Neopterin	Chimpanzee (*Pan troglodytes verus*)	free ranging	Taï National Park, Côte d’Ivoire	[57]
Neopterin	Bonobo (*Pan paniscus*)	free ranging	LuiKotale, Salonga National Park, Democratic Republic of the Congo	[58]
Neopterin	Gelada (*Theropithecus gelada*)	free ranging	Guassa Community Conservation Area, Ethiopia	[59]
Neopterin	Mandrill (*Mandrillus sphinx*)	free ranging	Lékédi Park, Gabon	[60]
suPar	Rhesus macaque (*Macaca mulatta*)	captive	German Primate Center, Germany	[61]
suPar	Rhesus macaque (*Macaca mulatta*)	free ranging	Cayo Santiago, Puerto Rico	[63]
IgA	Yellow baboon (*Papio cynocephalus*)	free ranging	Amboseli Baboon Research Project, Kenya	[66]
IgA	Sichuan golden monkey (*Rhinopithecus roxellana*)	captive	Shanghai Wildlife Zoo, China	[67]
IgA	Cynomolgus monkey (*Macaca fascicularis*)	captive	Bogor Agricultural University Primate Research Center, Indonesia	[68]
IgA	Chimpanzee (*Pan troglodytes schweinfurthii)*	free ranging	Gombe National Park, Tanzania	[69]
IgA	Barbary macaque (*Macaca sylvanus*)	semi-free ranging	Affenberg Salem, Germany	[70]
IgA	Red fronted lemur (*Eulemur rufifrons*)	captive and free ranging	Magdeburg Zoo, Germany and Kirindy Forest, Madagascar	[71]

### Future perspectives

**Fig. 4:**
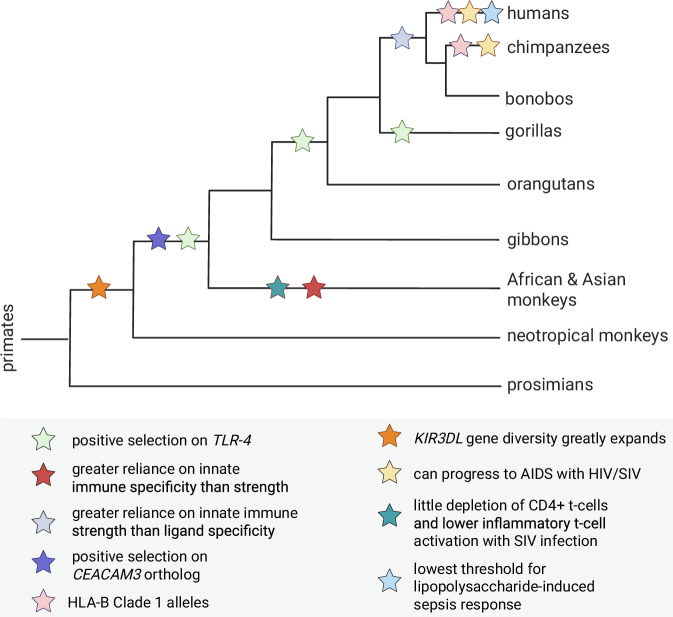
Visual summary of major lines of evidence examined in this review. This phylogenetic tree shows approximate evolutionary relationships between species, and colored stars each indicate a putative taxonomic difference [3, 12, 15, 16, 19, 39]. Branch lengths are not to exact scale and placement of stars along each branch do not reflect exact timing of that event. Created with BioRender.

Our review illustrates that while sufficient gaps remain in our understanding of the evolution of primate immunity due to lack of data from many species in the primate order, existing data provide intriguing evidence of variation in immune response that broadly occurs along taxonomic designations (Fig. 4). We propose that taxonomic differences in immune response and senescence with age may be informed by life history differences.

For example, positive selection on the TLR4 extracellular domain which recognizes lipopolysaccharide on Gram-negative bacteria [19], and evidence of increased reliance on generalized innate immune strength over specificity [15], suggest there may have been a wide variety of bacterial pathogens present in the great ape ancestral environment as a selective pressure [11, 12, 15, 19, 41]. However, great ape habitat range overlaps with that of many African monkeys, suggesting that additional factors warrant consideration. Great apes exhibit markedly prolonged periods of offspring development compared to other primates, which would increase early-life exposure to pathogens, including bacteria. A stronger innate immune response during development—especially one that has not been subject to immune senescence, which is known to be a broadly similar process in both aging macaque monkeys and humans—would thus require evolutionary adaptations for immune strength and improved TLR4 recognition [13, 21, 72]. It is well-established that human children have a much stronger innate immune response and rely on this generalized approach to fighting pathogens, rather than the specificity of adaptive immunity [73]. Perhaps the prolonged childhood that occurs in great apes, in comparison to other primates, has selected for strong innate immunity and repeatedly selected for improved TLR4 recognition.

To test this hypothesis, comparable patterns of immune aging should be confirmed in great apes to facilitate a useful comparison to rhesus macaque studies, as well as studying a greater variety of African and Asian monkeys. For example, an interesting great ape to study in this context would be the orangutan. They have the longest developmental period of all great apes, but are the least social [74], potentially reducing exposure to certain pathogens with improved transmission in high-density populations. A much larger dataset with species diversity that includes both functional genomic and life history data on immune response strategies could help to explain these specializations among great apes, especially in light of potential differences in patterns of age-related inflammation seen in two species of strepsirrhines versus in humans and macaques [47].

Recent advances in computational methods for detecting selection may offer additional insights—for example, methods which specifically detect directional selection in regulatory regions, like transcription factor binding sites, can highlight evolutionary constraints that differ from those in protein coding regions [75]. And, phylogeny-aware models like Ornstein–Uhlenbeck could be potentially useful for characterizing the evolution of immunity-related traits through evolutionary time in primates, as they have recently been applied to account for genetic variation within species which can mimic stabilizing selection towards the optimum within the model [76, 77]. Furthermore, TLR genetic loci under positive selection should also be functionally characterized in greater detail [11, 12, 19], whereby identifying the consequences of mutations in these TLRs within a phylogenetic context will provide clues to which pathogens exerted the greatest selective pressure on primate ancestral lineages. For example, this could potentially help resolve outstanding questions about positive selection on the *TLR4* gene regarding which gene regions are most important for Gram-negative bacteria recognition by innate immune cells. One study found the *TLR4* extracellular domain to be under strong positive selection starting in the lineage of all great apes [19], whereas another similar study, which looked at more parts of the gene beyond the coding region for the extracellular domain, identified positive selection within the lineage shared by both African and Asian monkeys and apes [12]. Resolving such questions could reveal specific mechanisms underlying strong innate immunity in great apes, and how this may relate to life history characteristics.

## Conclusion

Because humans are also great apes, the evolutionary history of our immune response strategies has enormous implications for treating human diseases. This has been an important consideration for many decades regarding HIV, but of course has broad potential for many other infectious and autoimmune diseases. There are now almost 500 named primate species, so the dearth of comprehensive data suitable for cross-species comparison is one of the largest obstacles facing the field, at present [9, 10]. Currently, it is not possible to speculate what level of taxonomic organization accounts for the most variation in immune response strategies in primates. However, as this review illustrates, novel research can be combined with existing knowledge regarding life history strategies among primates to guide new lines of inquiry. Obtaining data for all species across all areas of immunity research is obviously intractable. Throughout this review, we instead advocate for a practical approach to increasing comparative data across species, recommending increased species representation from certain phylogenetic lineages in specific areas of research that are most informative of phylogenetic.

**Glossary** **Acquired immunodeficiency syndrome (AIDS):** an SIV/HIV infection can cause progression to this often-fatal syndrome that can lead to widespread immune failure and secondary infections. This is known to occur in only a select few primate species, such as humans and some chimpanzees.

**Carcinoembryonic antigen-related cell adhesion molecule 3 (CEACAM3):** a receptor which helps neutrophils recognize dangerous gram-negative bacteria such as Haemophilus aegyptius, Helicobacter pylori, and Neisseria gonorrhoeae.

**Human immunodeficiency virus 1 (HIV-1) group M:** the pandemic strain of human immunodeficiency virus that represents over 90% of all HIV infections.

**Interleukin-1 receptor antagonist (IL-1Ra):** the interleukin-1 receptor antagonist is a member of the IL-1 family that binds to IL-1 receptors but does not activate a downstream response. Thus, it downregulates the inflammatory response by preventing proinflammatory IL-1 cytokine from binding there instead.

**Interferoninduced transmembrane proteins (IFITMs):** involved in the innate immune response to viruses, including globally-important viruses affecting humans like HIV-1 and SARS-CoV. They inhibit viral entry to host cells and viral replication.

**Immunoglobulin A (IgA):** an antibody that plays a role in the immune function of epithelial mucous membranes by protecting mucosal tissues from microbial invasion.

**Induced pluripotent stem cells (iPSC):** stem cells that can differentiate into a variety of cell types in the body, making them a renewable resource and useful for hard-to-obtain tissues.

**Killer Immunoglobulin-like Receptors (KIR):** employed by natural killer cells to recognize host cells and prevent cytotoxic action against the self. These receptors are encoded by the KIR3DL gene family, which is a diverse family of genes among primates.

**Major Histocompatibility Complex (MHC):** a highly polymorphic gene family which codes for cell-surface proteins that bind to fragments derived from foreign bacteria or viruses. MHC molecules are displayed on a cell’s surface to be recognized by T-cells receptors or KIRs.

**Massively parallel reporter assays (MPRA):** a next generation sequencing technique used to assess the regulatory activity of thousands of DNA sequences simultaneously, by “tagging” enhancer sequences of interest with a reporter gene (has a unique DNA barcode) to measure gene regulatory activity.

**Pathogen associated molecular patterns (PAMPs):** a broad term which encompasses the many types of extracellular patterns on invading pathogens that allow innate immune cells to recognize the pathogens as foreign.

**Simian/human immunodeficiency virus (SIV/HIV):** a species of retrovirus that infects helper T-cells in over 40 primate species.

**Soluble form of the urokinase plasminogen activator receptor (suPAR):** A cell-surface protein expressed mainly on immune cells and endothelial cells. The soluble form of this molecule is upregulated during immune activation. Thus, the concentration of suPAR is positively correlated with the degree of immune activation.

**Toll-like receptors (TLRs):** cell-surface ligands that recognize foreign molecular patterns on invading pathogens to help macrophages and other immune cells detect these pathogens.

**Tripartite motif-containing protein 5 (TRIM5):** an antiretroviral innate immune protein expressed on many mammalian cells that recognizes invading retroviruses by binding to the virus’ capsid that protects its viral core.

## Data Availability

This review did not generate original data, code, or reagents.
